# Women’s Social Empowerment and Gender Differences in Adults’ Cognitive Competences

**DOI:** 10.1093/geroni/igaa057.2445

**Published:** 2020-12-16

**Authors:** Daniela Weber

**Affiliations:** Vienna University of Economics and Business, Vienna, Wien, Austria

## Abstract

Female social empowerment has the potential to enhance women’s cognitive abilities. Our previous work investigating the role of gender equity in education and improved living conditions during early adulthood suggest that European women gain more from societal improvements over time than their male counterparts. This study extends this work by investigating the association between women’s social empowerment during childhood and gender differences in adults’ cognition for more than 30 OECD countries. We analyze established cognitive competence measures in literacy and numeracy with mixed effect models using the national survey data PIAAC collected within three rounds in 2011, 2014, and 2017. Our preliminary findings suggest that gender equity factors associated with women’s empowerment, are more beneficial for women’s cognitive key competences than men’s. High cognitive competences are particularly relevant at advanced age to enable an independent life and long economic activity.

